# Clinical evaluation of the diagnostic performances and treatment monitoring of the new PATHFAST TB LAM Ag assay in sputum specimens of patients with tuberculosis or with nontuberculous mycobacteria pulmonary disease

**DOI:** 10.1128/jcm.01269-25

**Published:** 2025-11-26

**Authors:** Theo Fouchet, Melissa Nait Chabane, Camille Allam, Zeina Awad, Faïza Mougari, Alain Hartmann, Murielle Rochelet, Emmanuelle Cambau

**Affiliations:** 1 AgroEcologie, UMR 1347, INRAE-UBE-IAD 117488, Dijon, France; 2 Service de mycobactériologie spécialisée et de référence, AP-HP Nord, site Bichat; Laboratoire associé du Centre National de Référence des Mycobactéries et de la Résistance aux Antituberculeux (CNR MyrMA), AP-HP 26930 , Paris, France; 3 IAME, Université Paris Cité, Inserm UMR1137, Paris, France; 4 Laboratoire de Biologie Médicale, Centre Hospitalier Francois Quesnay 37107 , Mantes-la-Jolie, France; The University of North Carolina at Chapel Hill School of Medicine, Chapel Hill, North Carolina, USA

**Keywords:** tuberculosis, LAM, diagnostic, treatment, antigen

## Abstract

Diagnosing pulmonary tuberculosis (TB) and monitoring treatment remain challenging. New tools such as the PATHFAST TB LAM assay (PHC Corporation, formerly LSI Medience Corporation, Tokyo, Japan; distributed by BioSynex, France; PF-LAM) may complement microscopy, culture, and NAAT. PF-LAM is an immunoassay quantifying lipoarabinomannan (LAM), a mycobacterial cell wall component, in sputum within 1 hour using an automated chemiluminescent reader. Diagnostic performance was first assessed on 100 sputum samples: 40 culture-positive for *Mycobacterium tuberculosis complex* (MTBC), 40 culture-positive for nontuberculous mycobacteria (NTM), and 20 culture-negative for both. We then tested 61 longitudinal sputum samples from 19 pulmonary TB patients under treatment. Four additional samples from one patient with NTM pulmonary disease (NTM-PD) were also tested. PF-LAM showed 75% sensitivity for MTBC detection, with a strong correlation between LAM concentration and culture time-to-positivity (Spearman ρ = 0.915, *P* < 0.0001). Specificity was 95% on culture-negative specimens. The test was also positive for 26/40 (65%) NTM-culture-positive samples. Notably, 19 of these 26 samples were obtained from NTM-PD patients, yielding 73% sensitivity for NTM-PD detection. The assay’s potential for treatment monitoring was demonstrated by significant negative correlations between LAM concentration and (a) treatment duration (ρ=–0.434, *P* = 0.0012) and (b) time to positive culture (ρ=–0.665, *P* < 0.0001). PF-LAM is a rapid and easy-to-use test for diagnosing pulmonary TB. While NTM cross-reactivity reduces specificity, it may provide diagnostic value for NTM-PD. Results on monitoring TB treatment are promising as no test is currently available for this indication.

**IMPORTANCE:**

Effective monitoring of the treatment response is essential for successful tuberculosis (TB) management as prolonged therapies require accurate evaluation to prevent relapse, treatment failure, or drug resistance. This study highlights the diagnostic and treatment-monitoring potential of the PATHFAST TB LAM Ag assay, which quantifies lipoarabinomannan (LAM) concentrations in sputum samples and correlates with bacterial load. For the first time, we also demonstrate the assay’s applicability for detecting and monitoring nontuberculous mycobacterial (NTM) pulmonary diseases, which are increasingly prevalent in industrialized countries. The semi-automated, rapid format (<17 minutes) of the PATHFAST TB LAM Ag assay provides a simple and reliable approach for assessing bacillary load during treatment, representing a promising tool for improving patient management and diagnostic efficiency in both TB and NTM-PD.

## INTRODUCTION

Tuberculosis (TB) is back as the leading cause of death from a single infectious agent (1.25 million attributed deaths/year) after the COVID-19 pandemic (1). The WHO estimated the TB burden as 10.8 million new cases in 2023, although three million are undiagnosed (1). These numbers were about the same in 2008, showing that the fight against TB is still a challenge in 2025. Transmission is mainly due to aerosols containing *Mycobacterium tuberculosis* complex (MTBC) produced by a patient with pulmonary TB (PTB) (2). This is why PTB diagnosis is important for global TB control.

TB diagnostic methods are mostly microbiological tools detecting MTBC presence by (i) acid-fast bacillus (AFB) visualization by direct smear microscopy, (ii) multiplication of bacteria in culture (liquid or solid media), and (iii) nucleic acid amplification techniques (NAAT) (3). These methods depend on the available laboratory resources and biosafety requirements inherent to class 3 respiratory pathogens (4). Recently, the TB diagnosis toolkit expanded to include the detection of the lipoarabinomannan (LAM) antigen in urine (5). LAM is a major cell wall component released from active MTBC cells, excreted through the urine. Urinary LAM is predominantly detected in disseminated or advanced TB cases (5, 6), although positivity was also reported in patients living with HIV with nondisseminated pulmonary disease (7). It is recommended by the WHO for persons living with HIV and suspected of TB (8).

LAM detection in urine is available as an immunochromatography point-of-care test (6) and was once tested in sputum with an in-house research-use ELISA test, bringing promising results without any further clinical studies (9). The PATHFAST TB LAM Ag assay (PHC Corporation, formerly LSI Medience Corporation, Tokyo, Japan; distributed by BioSynex, France; PF-LAM) is a new commercially available test to detect and measure LAM in sputum, using a chemiluminescent assay with automated reading. A preliminary study reported its good analytical performances on artificial samples and first assessment on a batch of biobank samples (10). The test was also applied to stored sputum samples from early bactericidal activity trials, demonstrating its ability to monitor the decrease in bacterial burden during treatment (11).

We conducted a clinical evaluation of the PF-LAM by testing 100 samples from patients suspected or diagnosed with PTB or with pulmonary disease due to nontuberculous mycobacteria (NTM-PD). We then included longitudinal samples obtained from PTB patients under treatment, seeking if antigen testing can monitor the microbiological burden. PF-LAM test results analyzed with regard to those obtained with standard tools showed good clinical performances in both the diagnosis and monitoring of patients during treatment.

## MATERIALS AND METHODS

This study was conducted in accordance with the 2015 STARD guidelines (12).

### Clinical sputum samples

One hundred sputum samples received from individuals suspected of PTB or NTM-PD (four from 2019, five from 2020, one from 2022, fifteen from 2023, and 76 from 2024) were taken for PF-LAM performance evaluation. For sensitivity assessment, 40 were selected from patients with MTBC-positive cultures prior to the initiation of anti-tuberculosis treatment, and according to smear microscopy results: 20 samples smear-negative, four with <1 AFB per field, seven with 1-9 AFB/field, five with 10-100 AFB/field, and four with >100 AFB/field. In addition, we tested 40 samples (20 smear-positive and 20 smear-negative) culture-positive for NTM. NTM species were identified using line probe assays (GenoType CM/AS and/or NTM-DR, Hain Bruker, Massachusetts, USA), MALDI-TOF (Bruker, Massachusetts, USA), or with Sanger sequencing of the *hsp65* gene when necessary (3).

To evaluate assay specificity, we tested 20 additional sputum samples with negative results for smear microscopy and culture for MTBC and NTM.

To assess PF-LAM potential for monitoring the treatment response, based on their availability, serial sputum specimens from 19 PTB patients undergoing anti-tuberculosis therapy at various time points (from day 0 and up to 56 days) were selected. We also tested follow-up samples from one patient diagnosed with NTM-PD and undergoing anti-NTM therapy.

Samples were processed in standard care conditions in our clinical microbiology laboratory. Briefly, sputum samples were liquefied, decontaminated, and centrifuged, and the pellet was resuspended in 3 mL of buffer using the NAC-PAC kit (AlphaTec, Vancouver, USA) and analyzed according to standard diagnostic procedures described previously (3). Auramine-stained smear microscopy for AFB detection, solid culture on Coletsos medium (an egg-based medium supplemented with sodium pyruvate [3] [BioRad]), liquid culture using the Mycobacteria Growth Indicator Tube (MGIT) system (Becton Dickinson, Franklin Lakes, NJ, USA), and molecular testing using the Xpert MTBC/RIF Ultra assay (Cepheid, Sunnyvale, CA, USA) were performed. A sample was considered positive at culture if at least one culture medium, MGIT or Coletsos, yielded MTBC or NTM growth.

### PATHFAST TB LAM Ag assay

The PF-LAM was done following the manufacturer’s recommendations. Calibrators and quality controls were tested monthly prior to the clinical samples.

Calibrators were two manufacturer-provided standards of undisclosed low and high LAM concentrations within the assay detection range (10–50,000 pg/mL). They were used to generate a two-point standard curve. Two quality control samples were provided by the manufacturer (QC1: 70–130 pg/mL; QC2: 25,000–35,000 pg/mL) to monitor assay accuracy and precision.

A short run of analytical validation was also conducted for assessing the repeatability and reproducibility, with five analytical runs performed per day over 3 consecutive days, using the same lot of reagents. For each run, both QC1 and QC2 concentrations were measured in duplicate. We calculated mean, standard deviation (SD), and coefficient of variation (CV) of LAM concentrations for each run and each day to assess the inter- and intra-variability of the test. According to the standard of care conditions for semi-quantitative immunoassays on complex biological matrix, the assay was considered precise if the CV remained below 20%.

The PF-LAM is a two-step process with LAM manual extraction followed by automated LAM quantification. LAM extraction was performed by incubating 200 µL of liquefied-decontaminated and centrifuged sputum samples with 100 µL of 1.0N NaOH (Merck KGaA, Darmstadt, Germany) during 20 minutes at 100°C. Samples were then neutralized with 50 µL of 5 M NaH₂PO₄ (Merck KGaA, Darmstadt, Germany) and centrifuged at 3,000 *g* for 5 minutes at room temperature. The supernatant was collected as the LAM extract, and a 100 µL volume was transferred into a ready-to-use cartridge loaded into the PATHFAST analyzer (10). The LAM concentration was measured within 15 minutes and expressed as pg/mL. The lower and upper limits of quantitation were previously calculated as 10 pg/mL and 50,000 pg/mL, respectively (10). Each sample with LAM concentration >50,000 pg/mL was diluted 1:10 to calculate the exact LAM concentration.

### Statistical analysis

All statistical analyses were performed with RStudio software. (R version 4.3.1, 2023-06-16 ucrt). A *P*-value < 0.05 was used for all tests of statistical significance.

## RESULTS

### Analytical precision of the PATHFAST LAM assay

Although the precision of the assay has previously been reported (10), an independent validation was performed to confirm reproducibility in our setting. Results of intra- and inter-assay variability are summarized in Table S1. Mean LAM concentrations for QC 1 and QC 2 were 99.0 pg/mL and 33,666 pg/mL, respectively. Intra-assay CVs ranged from 5.6% to 9.9%, and inter-assay CVs ranged from 6.4% to 10.7%, which is consistent with data reported by PHC Corporation (10)(Table S1).

Mean measured LAM concentrations for both quality controls were within the ranges of acceptable values, supporting the analytical reliability of the PF LAM in our setting (Table S1).

Overall, these results demonstrate excellent analytical accuracy and confirm the reproducibility of the results under the operating conditions of our laboratory.

### Diagnostic performances for MTBC detection

PF-LAM results were analyzed according to results of culture, Xpert MTB/RIF Ultra, and microscopy. As shown in Table 1, a sensitivity of 75% (30/40, 95% CI:58.8–87.3), a specificity of 95% (19/20, 95% CI:75.1–99.9), and an overall agreement of 81.7% (49/60, 95% CI:69.6–90.5) were found. According to our sample selection, PPV and NPV were 96.8% (30/31, 95% CI: 83.3–99.9) and 65.5% (19/29,95% CI: 45.7–82.1) respectively (Table 1). Cochran’s Q test revealed a significant difference in sensitivity among the three methods (*P* < 0.05). McNemar’s tests showed significant differences in sensitivity between microscopy and Xpert MTB/RIF Ultra, as well as between microscopy and the PF-LAM (*P* < 0.01), while no significant difference was found between Xpert MTB/RIF Ultra and PF-LAM (*P* = 0.22). No significant differences in specificity were observed among the three groups (*P* = 0.36). Regarding overall agreement, significant differences were observed between microscopy and Xpert MTB/RIF Ultra and between PF-LAM and microscopy (*P* < 0.05), with no significant difference between PF-LAM and Xpert MTB/RIF Ultra (*P* = 0.14).

**TABLE 1:** Sensitivity and specificity of the PATHFAST TB LAM Ag assay (PF LAM) for the detection of *Mycobacterium tuberculosis* complex in sputum^*f*^

Assay	Sensitivity *n*/*n* (%) 95% CI^*a*^			Specificity *n*/*n* (%) (95% CI)^*b*^	Overall agreement *n*/*n* (%) (95% CI)^*c*^	PPV *n*/*n* (%) (95% CI)^*d*^	NPV *n*/*n* (%) (95% CI)^*e*^
	SM+C+	SM-C+	Total C+	C-	All		
PF LAM	20/20 (100%) 83.2–100	10/20 (50%) 27.2–72.8	30/40 (75%) 58.8–87.3	19/20 (95%) 75.1–99.9	49/60 (81.7%) 69.6–90.5	30/31 (96.8%) 83.3–99.9	19/29 (65.5%) 45.7–82.1
Xpert MTB/RIF	20/20 (100%) 83.2–100	15/20 (75%) 50.9–91.3	35/40 (87.5%) 73.2–95.8	20/20 (100%) 83.2–100	55/60 (91.7%) 81.6–97.2	35/35 (100%) 90–100	20/25 (80%) 59.3–93.2
Smear microscopy	20/20 (100%) 83.2–100	0/20 (0%) 0–16.8	20/40 (50%) 33.8–66.2	20/20 (100%) 83.2–100	40/60 (66.7%) 53.3–78.3	20/20 (100%) 83.2–100	20/40 (50%) 33.8–66.2

^*a*^ No. of assay-positive samples/no. of TB-positive samples (SM- and culture-positive plus SM-negative and culture-positive samples).

^*b*^ No. of assay-negative samples/no. of TB-negative samples.

^*c*^ No. of assay-positive samples/no. of TB-positive and -negative samples.

^*d*^ No. of true TB-positive samples/no. of assay-positive samples (true-positive and false-positive samples).

^*e*^ No. of true TB-negative samples/no. of assay-negative samples (true-negative and false-negative samples).

^*f*^ SM+C+: smear microscopy positive- and culture-positive patients, SM-C+: smear microscopy negative and culture positive patients, C+: MTBC-positive samples, C-: negative samples. PPV, positive predictive value; NPV, negative predictive value; CI, confidence interval.

**Fig 1:**
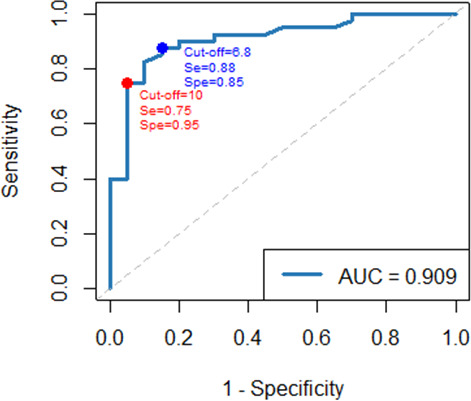
Receiver operating characteristic (ROC) curve obtained from the results of the PATHFAST TB LAM Ag assay for the 40 TB-positive patients and the 20 TB-negative patients. The dashed line marks the curve of a random classifier. The red dot and the blue one correspond to the cut-off value chosen for this study and to the optimal cut-off value according to Youden's index, respectively. Se: sensitivity; Spe: specificity; AUC; area under the curve.

A receiver operating characteristic (ROC) curve was generated based on LAM concentrations across all samples (Fig. 1). The area under the curve (AUC) was 0.909, indicating a high diagnostic accuracy. At the predefined cut-off of 10 pg/mL based on Akinaga et al*.* (10), sensitivity and specificity were 75.0% and 95.0%, respectively, which are the same values as those calculated in Table 1. Notably, the optimal threshold calculated using Youden’s index from our data set was 6.8 pg/mL, suggesting a slightly lower cut-off value than previously reported (10) (Fig. 1).

**Fig 2:**
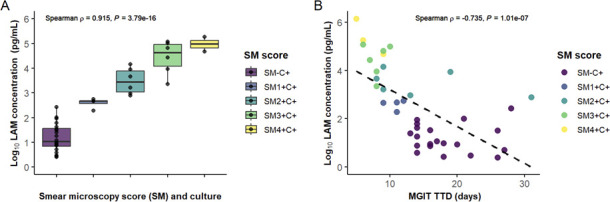
Relationship between LAM concentrations determined by the PATHFAST TB LAM Ag assay and MTB bacterial load, evaluated by smear positivity (score 1+ to 4+) and time to detection (TTD) of liquid MGIT culture. (**A**) Positive correlation of LAM concentration with regard to five categories defined by the smear microscopy score and liquid culture result. (**B**) Negative correlation of LAM concentration with TTD of MGIT. SM, smear microscopy; C. culture; TTD, time to detection.

LAM results were categorized according to bacterial load, estimated using smear microscopy scores and MGIT time to detection (TTD), as shown in Fig. 2. There were significant positive associations between LAM concentration and bacterial load categories (Fig. 2A, Spearman’s rank correlation coefficient of 0.915 [95% CI: 0.8320.951, *P* < 0.001]) and negative associations between LAM levels and TTDs (Fig. 2B, Spearman’s ρ = −0.735 [95% CI: −0.89; −0.508, *P*-value < 0.001]).

### Evaluation of the LAM concentration dynamics during anti-tuberculosis treatment

**Fig 3:**
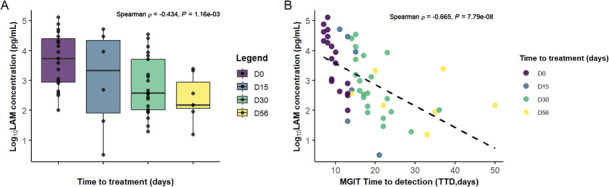
Relationship between LAM concentrations determined by the PATHFAST TB LAM Ag assay and *Mycobacterium tuberculosis* complex bacterial load. (**A**) Evolution of PF-LAM concentrations (log10) over grouped treatment time points baseline (Day 0), Days 1–15 (D15). Days 16–30 (D30), and Days 31–56 (D56). (**B**) Relationship between PF-LAM concentrations (log10) and time to positivity detection in MGIT, during anti-tuberculosis treatment (n = 19 patients). A negative correlation was observed between LAM concentration and time to detection. TTD, Time to detection in days.

The potential of PF-LAM as a tool for monitoring anti-tuberculosis treatment was assessed on sputum from 19 patients at various time points of treatment up to 56 days (from 2 to 5 samples by patient). The 53 samples were grouped in four different timelines of treatment: baseline (Day 0, D0), D1 to D15, D16 to D30, and after D31 up to D56. A decrease in LAM concentration was observed across the four timelines samples (Fig. 3A). A Spearman correlation test showed a moderate but statistically significant negative correlation between treatment duration and LAM concentration (ρ=–0.434, *P* = 0.00116). A statistically significant negative correlation was also observed (ρ=–0.665, *P* < 0.0001) between LAM concentration and TTD, showing that lower LAM levels are associated with delayed culture positivity and with reduced bacterial burden (Fig. 3B).

**Fig 4:**
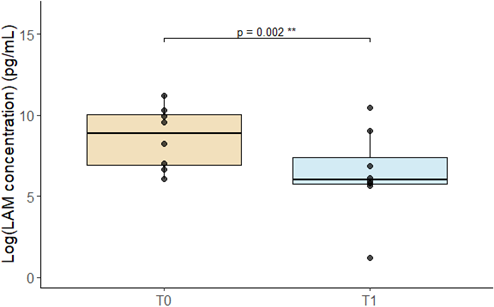
Decrease in LAM concentration determined by the PATHFAST TB LAM assay between Day 0 and after 1 month of anti-tuberculosis therapy in eight patients with paired samples. TO: day 0 prior to initiation of anti-tuberculosis treatment; T1: day around 1 month after initiation of anti-tuberculosis treatment.

We further conducted a focused analysis on a subset of eight patients with paired sputum samples available both at baseline (T0) and after 1 month (T1) of treatment. Using a linear mixed model (LMM), we confirmed a significant decrease in LAM concentrations after 1 month of treatment (Fig. 4, *P* = 0.002).

For one more patient who experienced clinical relapse episodes, we had collected seven samples over more than 160 days. After a first decrease due to the treatment, we observe indeed an increase in LAM concentration concordant with MTBC culture-positive samples. The corresponding data are presented in Fig. S1.

### PF-LAM results for sputum samples with NTM-positive culture

PF-LAM was investigated for 40 samples culture-positive for NTM (20 smear-positive and 20 smear-negative), with eight slow-growing and five rapid-growing NTM species. Results are detailed in Table 2.

**TABLE 2:** Positivity of the PATHFAST TB LAM Ag (PF-LAM) assay (n, %) on sputum samples culture-positive for nontuberculous mycobacteria (NTM)

NTM^*a*^ species	Growth rate	No. tested	PF-LAM positive *N* (%)
*M. abscessus*	Rapid	5	1 (20%)
*M. chelonae*	Rapid	3	3 (100%)
*M. fortuitum*	Rapid	2	1 (50%)
*M. peregrinum*	Rapid	1	0
*M. senegalense*	Rapid	1	0
*M. chimaera*	Slow	9	7 (78%)
*M. avium*	Slow	6	5 (83%)
*M. xenopi*	Slow	5	3 (60%)
*M. paragordonoae*	Slow	3	1 (33%)
*M. intracellulare*	Slow	2	2 (100%)
*M. haemophilum*	Slow	1	1 (100%)
*M. colombiense*	Slow	1	1 (100%)
*M. septicum*	Slow	1	1 (100%)
Total		40	26 (65 %)

^*a*^ NTM, nontuberculous mycobacteria.

Out of 40, 26 samples (65%) yielded a positive PF-LAM result. The PF-LAM test yielded positive results for all NTM species, except *M. peregrinum* and *M. senegalense* (Table 2). Among these positive samples, 19 were from patients diagnosed with NTM-PD: 14 infected with *M. avium* complex (*M. avium*, *M. intracellulare*, *M. chimaera*, and *M. colombiense*), three with *M. xenopi*, one with *M. abscessus,* and one with *M. haemophilum*. The remaining seven positive samples were as follows: one from a patient diagnosed with TB (smear-positive sample), but the culture was contaminated with *M. chelonae*, one from a patient suspected of TB but culture-negative, and five patients not diagnosed for mycobacterial infection. For the latter five samples, all were smear-negative and grew NTM species rarely involved in pulmonary infection (13), such as *M. fortuitum* complex, *M. chelonae,* and *M. paragordonae*.

**Fig 5:**
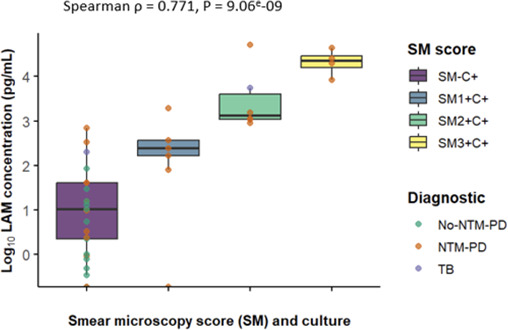
Relationship between LAM concentrations determined by the PATHFAST TB LAM Ag assay and bacterial load of nontuberculous mycobacteria infection (NTM). Positive correlation of LAM concentration with smear microscopy and liquid culture categories. SM, smear microscopy; C, culture; TTD, time to detection; NTM-PD, diagnostic of NTM pulmonary infection; No-NTM-PD, no diagnosis of NTM-PD; TB, patient with ancient pulmonary tuberculosis.

Sensitivity for detecting NTM-PD was overall 73% (19/26) since 26 samples NTM culture-positive were from patients diagnosed with NTM-PD (15 smear-positive and 11 smear-negative) according to guidelines criteria (14), and 19 were PF-LAM-positive. A strong positive correlation was observed between LAM levels and smear scores (Spearman’s ρ = 0.771, 95% CI:0.541–0.888, *P* < 0.001), as shown in Fig. 5.

**Fig 6:**
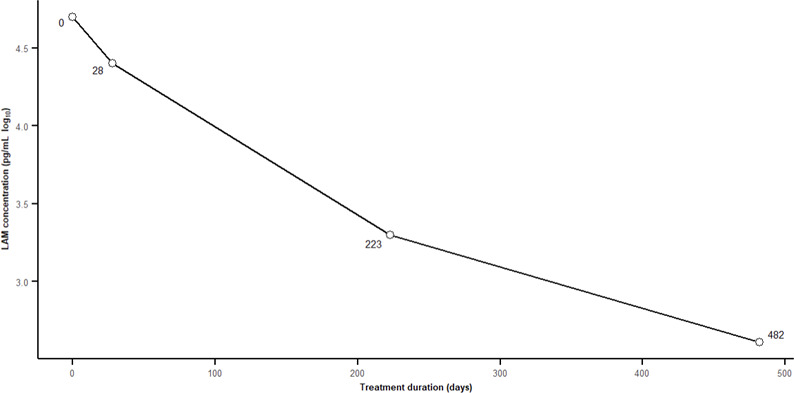
Evolution of LAM concentration during anti-NTM treatment of a patient with NTM-PD (*M. avium*). LAM was dosed at Day 0, Day 28, Day 223, and Day 482.

As it has been done for TB monitoring, we evaluated one patient with *M. avium* NTM-PD follow-up samples at baseline (Day 0), D28, D223, and D482 after initiation of treatment. As illustrated in Fig. 6, LAM levels declined by approximately 1.5 log over the treatment course.

Overall, the sensitivity of PF-LAM for mycobacteria detection was 70% (56/80) based on these previous 40 MTB samples and the 40 NTM samples.

## DISCUSSION

Microbiological diagnosis of tuberculosis currently relies on smear microscopy, NAAT, and culture, depending on available resources. Antigen detection is only used in urine (WHO recommendations for persons living with HIV suspected of TB [8, 15]) but has not been used so far in clinical practice on sputum of patients with PTB. We showed that a standardized test measuring lipoarabinomannan (LAM) concentrations in sputum can be used as a new diagnostic tool for PTB. This is the first commercially available test able to monitor the decrease in MTB burden in patients following antituberculosis treatment.

LAM is a major glycolipid component of the mycobacterial cell wall, and its detection in urine is a biomarker correlated with TB in immunocompromised individuals such as HIV-positive patients (8, 15, 16). Currently endorsed tests include the Determine TB-LAM Ag assay (Abbott), recommended for HIV-positive patients with suspected TB (disseminated or not), although it suffers from limited sensitivity (17). SILVAMP TB-LAM (FujiLAM) reported sensitivities from 40% to 87% depending on the population and clinical setting. It is not WHO-endorsed, due to large variability among lots (5, 18). These tests are, however, not recommended for sputum testing and treatment monitoring (17, 19).

Very few studies investigated LAM detection in either HIV-negative individuals, sputum for PTB, or tracking treatment response (9). To fill this gap, PF-LAM was developed as a commercial product, semi-automated, chemiluminescent immunoassay system, designed to quantify LAM concentrations in sputum specimens. Preliminary studies (10, 11) showed good analytical results and correlation with bacterial load; recent data also support its use for TB treatment monitoring (20).

Unlike previous studies, which primarily assessed analytical performance or feasibility (9–11, 20), our work evaluated PF-LAM in a clinically ascertained cohort including both MTB- and NTM-positive patients and examined longitudinal LAM dynamics during treatment.

We first analyzed clinical sputum samples with PF-LAM at the diagnostic step, and the results were concordant with those of smear microscopy, Xpert MTB/RIF Ultra, and culture data. Clinically, the assay achieved 75% sensitivity (30/40) and 95% specificity (19/20) for the detection of MTBC. These results are consistent with preliminary ones obtained with both the PATHFAST platform (10) and LAM-targeting ELISAs (9). LAM concentration correlated with MGIT TTD, but its correlation with colony counts on solid media was not assessed since these results are only semi-quantitative and not reliable above 50 colonies. This supports the hypothesis that LAM concentration in sputum may serve as a dynamic marker of bacillary burden.

Our results also showed evidence that LAM measurement in sputum by PF-LAM could be used as a dynamic marker for treatment response. Culture is still the gold standard for assessing treatment efficacy and has major drawbacks: takes several weeks to get the result, requires biosafety level 3 infrastructure, and is susceptible to contamination with consequently no result for culture, eventually. NAATs such as Xpert MTBC/RIF Ultra offer fast results but were not recommended for the TB follow-up since they can remain positive when the culture is negative (21, 22). Emerging viability-PCR methods, such as MBLA (23), have shown promise but often require advanced laboratory setups, longer processing times, and ultra-sensitive detection systems (24–26). Other antigens or biomarkers, such as PT64-release (27), were also tested but without available tests (23, 27). The simplicity, automation, standardization, and short turnaround time (17 minutes) are advantages of PF-LAM.

There are some limitations in our study. First, the study is retrospective, relying on a relatively small number of samples from TB-positive and TB-negative patients, which is few with regard to the thousands we analyze yearly in routine conditions. Patient-level clinical data for culture-negative samples could not be provided due to IRB restrictions, so undiagnosed mycobacterial disease cannot be excluded. Treatment monitoring was only assessed up to 56 days, whereas clinically relevant follow-up occurs at 3 and 6 months; post-treatment sampling beyond day 0 is not routinely performed in standard-of-care practice (https://www.who.int/publications/i/item/9789240107243). The fact that we worked on samples kept frozen is not a limitation since a good correlation between LAM measurements in frozen and fresh sputum was recently reported (11). The treatment monitoring investigation was also based on a small number of patients. The majority of samples were, however, collected during the first month of therapy, a period when the decline in MTBC load is the most important (28, 29). To address these limitations, a larger-scale prospective study should be conducted, with several months of monitoring and including clinical outcomes such as culture conversion, treatment failure, and relapse. Cost-effectiveness, operational feasibility, and implementation aspects remain to be explored, particularly in high-burden, resource-constrained settings where automation and turnaround time could play a critical role in adoption. Nevertheless, although semi-automated, PF-LAM currently requires manual inactivation and extraction, including heating and centrifugation, making it a laboratory-based assay; future simplifications could enable point-of-care use.

The PF-LAM was also positive in patients with NTM-PD, whether with rapid- or slow-growers NTM. Cross-reactivity between MTBC and NTM is well known for many assays including ribosomal gene amplification, serology, and antigen testing (28, 29). While this limits PF-LAM specificity for TB in high-income settings with rising NTM prevalence, it may also support its use in the diagnosis of NTM pulmonary infections when culture is delayed or unavailable. This test might also be used for the monitoring of NTM-PD treatment, which would be very useful since these patients are positive for many months or years and require regular monitoring to assess the efficacy of non-optimized antimicrobial therapy, especially for cases due to *M. avium* complex and *M. abscessus* (14).

In conclusion, these findings support the potential role of the PATHFAST TB LAM Ag assay as a tool not only for diagnosis but also for treatment monitoring of pulmonary mycobacterial infections. In this indication, no tests are currently available as a clinical microbiology marketed assay.

## SUPPLEMENTAL MATERIAL

Table S1 and Figure S1 jcm.01269-25-s0001.docx Table S1 : Inter- and intra-day detection of LAM in quality controls. Figure S1 : LaM concentration during TB treatment of a patient with clinical relapse episodes.

ASM does not own the copyrights to Supplemental Material that may be linked to, or accessed through, an article. The authors have granted ASM a non-exclusive, world-wide license to publish the Supplemental Material files. Please contact the corresponding author directly for reuse.
