# Supplementary material for: Clinical evaluation of the diagnostic performances and treatment monitoring of the new PATHFAST TB LAM Ag assay in sputum specimens of patients with tuberculosis or with nontuberculous mycobacteria pulmonary disease
Source: J Clin Microbiol. 2025 Nov 26;63(12):e01269-25. doi: 10.1128/jcm.01269-25 (PMC12710305; doi:10.1128/jcm.01269-25)
Supplement: Table S1 and Figure S1 — Table S1 : Inter- and intra-day detection of LAM in quality controls. Figure S1 : LaM concentration during TB treatment of a patient with clinical relapse episodes. [file jcm.01269-25-s0001.docx]

**Supplementary data**

**Table S1**

Inter- and intra-day detection of LAM in quality controls, QC1 and QC2, assessing precision performances

| Sample | Mean (pg/mL) | Repetability | | Reproductibility | |
| --- | --- | --- | --- | --- | --- |
|  |  | SD (pg/mL) | CV (%) | SD (pg/mL) | CV (%) |
| QC 1 | 99.0 | 9.7 | 9.9 | 10.6 | 10.7 |
| QC 2 | 33 666 | 1891 | 5.6 | 2147 | 6.4 |

SD, Standard deviation; CV, coefficient of variation; QC 1 and 2, Quality control 1 and 2


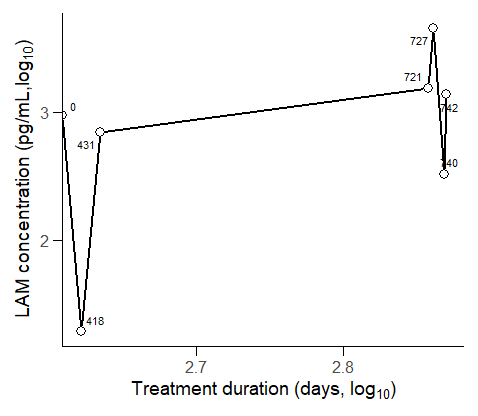


**Fig. S1**. Evolution of LAM concentration during anti-TB treatment of a patient with clinical relapse episodes. LAM was dosed at Day 0, Day 418, Day 431, Day 721, Day 727, Day 740 and Day 742.
